# Accumulation of Rhodopsin in Late Endosomes Triggers Photoreceptor Cell Degeneration

**DOI:** 10.1371/journal.pgen.1000377

**Published:** 2009-02-13

**Authors:** Yashodhan Chinchore, Amitavo Mitra, Patrick J. Dolph

**Affiliations:** Department of Biology, Dartmouth College, Hanover, New Hampshire, United States of America; Harvard Medical School, Howard Hughes Medical Institute, United States of America

## Abstract

Progressive retinal degeneration is the underlying feature of many human retinal dystrophies. Previous work using *Drosophila* as a model system and analysis of specific mutations in human rhodopsin have uncovered a connection between rhodopsin endocytosis and retinal degeneration. In these mutants, rhodopsin and its regulatory protein arrestin form stable complexes, and endocytosis of these complexes causes photoreceptor cell death. In this study we show that the internalized rhodopsin is not degraded in the lysosome but instead accumulates in the late endosomes. Using mutants that are defective in late endosome to lysosome trafficking, we were able to show that rhodopsin accumulates in endosomal compartments in these mutants and leads to light-dependent retinal degeneration. Moreover, we also show that in dying photoreceptors the internalized rhodopsin is not degraded but instead shows characteristics of insoluble proteins. Together these data implicate buildup of rhodopsin in the late endosomal system as a novel trigger of death of photoreceptor neurons.

## Author Summary

Irreversible loss of photoreceptor cells has been attributed as a cause of blindness in many retinal degenerative disorders. One such group of disorders is retinitis pigmentosa, which affects 1 in 3,000 individuals. Over 100 mutations in the light-sensing molecule rhodopsin have been identified in patients with autosomal dominant retinitis pigmentosa. These mutations affect rhodopsin transport to the outer segments of rod photoreceptor cells, rhodopsin folding, and rhodopsin endocytosis. In *Drosophila* photoreceptors, endocytosis of a large amount of rhodopsin at a rapid rate results in cell death. To further understand the role of endocytosis in triggering cell death, we used previously characterized mutants in which lysosomal degradation is compromised. We show that retinal degeneration can also be induced in these genetic backgrounds after rhodopsin is endocytosed, suggesting that failure to degrade internalized rhodopsin in a timely manner triggers cell death of photoreceptor neurons. We also present direct cellular biological evidence of rhodopsin accumulation in the cell bodies of photoreceptors, only in mutant backgrounds that undergo retinal degeneration. We could rescue the degeneration by preventing rhodopsin endocytosis and accumulation. Thus, our results indicate the vital role of lysosomal turnover of rhodopsin in maintaining photoreceptor viability.

## Introduction

Inherited retinal degenerative disorders in humans exhibit heterogeneity in their underlying causes and clinical outcomes [1]. Diverse causes have been attributed, including disruption of genes that are involved in phototransduction, biosynthesis and folding of the rhodopsin molecule, and the structural support of the retina. However, a clear understanding of the mechanism of photoreceptor cell death has yet to be worked out. The *Drosophila* phototransduction pathway, mediated by the major rhodopsin (Rh1), has served as a model system for studying retinal degeneration [2]–[4]. Light absorption by Rh1 triggers a signaling pathway leading to the activation of an eye-specific phospholipase C encoded by the *no-receptor potential A (norpA*) locus and this is essential for the photoresponse, and eventually the opening of cation-specific ion channels.

Like many other G protein-coupled receptors, Rh1 undergoes endocytosis following activation [5],[6]. Perturbation of endocytic regulation of Rh1 has deleterious effects on photoreceptor cell physiology. This has been well documented for *Drosophila norpA* mutants. In *norpA* flies, persistent complexes between rhodopsin and arrestin are formed due to a block in light-triggered Ca^2+^-dependent phosphorylation of Arr2. Arr2 then recruits the endocytic machinery triggering massive internalization of Rh1, resulting in light-dependent retinal degeneration [7],[8]. Pathogenic endocytosis of Rh1 is also demonstrated in other phototransduction mutants of *Drosophila* such as *retinal degeneration C (rdgC)* [9] and *arrestin1 (arr1)* [6]. Interestingly, formation of toxic Rhodopsin-Arrestin complexes is also reported for mutants of human rhodopsin associated with severe forms of Autosomal Dominant Retinitis Pigmentosa (ADRP) [10],[11]. For example, mutations at Arg135 are associated with severe forms of retinitis pigmentosa and exhibit a high affinity for arrestin, undergo endocytosis, and display endosomal abnormalities. These instances underscore the importance of studying Rh1 endocytosis and its relation to the photoreceptor health.

Earlier work has indicated that Rh1 internalization plays a crucial role in *norpA* and *rdgC*-mediated photoreceptor cell death [9],[12],[13]. However the role that is played by downstream endocytic trafficking, if any, has not been addressed. Here, we investigate the effect of post-endocytic modulation of Rh1 trafficking in influencing retinal degeneration in *Drosophila*. We show that the granule group mutants that have impaired lysosomal delivery underwent light-dependent retinal degeneration in an otherwise wild type background. In *norpA* as well as granule group mutants, Rh1 accumulated in the Rab7-positive late endosomes as persistent vesicles. Preventing Rh1 accumulation by vitamin A deprivation (which reduces the total Rh1 amount) or by using *Rh1Δ356* (an Rh1-variant that cannot be endocytosed) rescued photoreceptor cell death in granule group mutants. We also observe that, in *norpA*, the internalized Rh1 is not degraded. Taken together, our results indicate the vital role of lysosomal turnover of Rh1 in maintaining photoreceptor viability.

## Results

### Functional Late Endosomal System Is Essential for Photoreceptor Cell Viability after Rh1 Internalization

**Figure 1:**
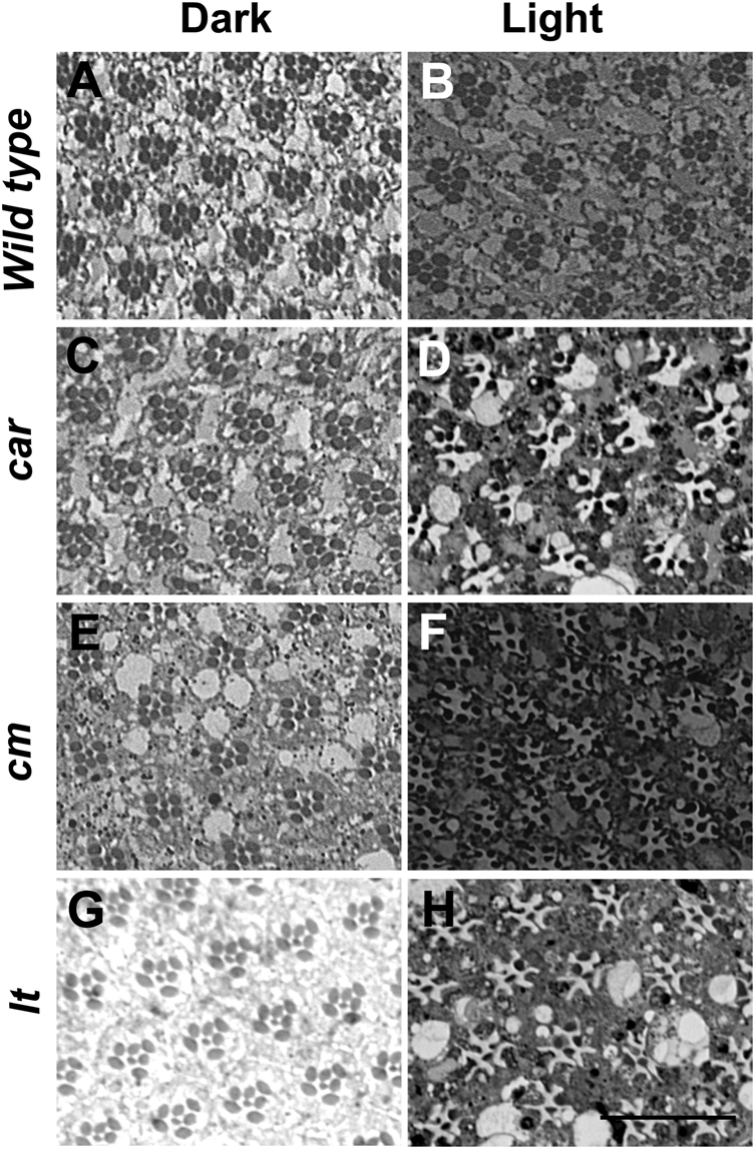
Mutations affecting trafficking to the lysosomes result in light-dependent retinal degeneration. Cross sections (1 µm) of retinas from white-eyed wild type, *car*, *cm*, and *lt* flies. Respective genotypes were dark-reared (A, C, E and G) or exposed to continuous room light for 7 days (B, D, F and H) prior to fixation. Retinal degeneration is only observed in light-exposed flies. Tissues were fixed and embedded as described in Materials and Methods. Scale bar, 20 µm.

Previous work on *Drosophila norpA* and *rdgC* mutants has revealed that massive endocytosis of Rh1 following light-exposure is the underlying cause of photoreceptor cell death [9],[12]. To better understand the relationship between endocytosis and cell death we examined previously characterized mutants believed to affect late endosome trafficking/lysosome biogenesis in *Drosophila*. These genes which belong to the so called “granule group” play an important role in lysosome biogenesis and function [14]–[17]. A mutation in the *carnation* (*car*) gene, which is involved in late endosome to lysosome trafficking [17], displays light-dependent retinal degeneration in an otherwise wild type background (Figure 1D). The *car* gene product is the *Drosophila* homolog of yeast Vps33p, which is part of the Class C Vacuolar Protein Sorting (VPS) protein complex. VPS-C complex is involved in fusion of endosomes with vacuoles, which are equivalent to metazoan lysosomes [18]. Since, dark-raised *car* flies have retinal morphology similar to dark or light-raised wild type flies (Figure 1A–C), we hypothesized that this degeneration was due to defects in Rh1 degradation.

To test if the degeneration was a result of defective lysosome function we used a mutation called *carmine* (*cm*). The *cm* flies are defective in the medium chain subunit of the adaptor protein complex AP-3, which is necessary for transport to the pigment granules- the lysosome-related organelles [14],[15],[19]. AP-3 is involved in the delivery of Golgi-derived vesicles to the vacuoles in yeast [20]. Dark-raised *cm* flies resembled wild type in terms of normal retinal morphology. We could not identify any visible adverse effect on the retinal morphology and photoreceptor development (Figure 1E). Continuous light exposure of *cm* flies resulted in retinal degeneration (Figure 1F) indicating that lysosomal function plays an important role in viability of photoreceptor neurons in light.

To further explore the role of granule group genes in retinal degeneration, we investigated the effect of mutation in the *light* gene, which encodes a homolog of yeast Vps41p. Vps41p interacts with class C VPS complex and with another protein Vps39p, forms an active HOPS (Homotypic vacuole fusion and Protein Sorting) complex that functions as an effector of Ypt7/Rab7 [21],[22]. Furthermore, Vps41p has been shown to interact with the AP-3 adaptor complex in yeast [23]. Like other granule-group mutants, *lt* flies had normal eye morphology and did not show any signs of degeneration in constant darkness (Figure 1G). Mutation in the *lt* gene rendered flies susceptible to light-induced retinal degeneration (Figure 1H). Thus, these three lines of evidence show that interference with lysosomal function during light-exposure results in photoreceptor cell death.

### Rh1 Endocytosis and Its Buildup Is Required for Cell Death

**Figure 2:**
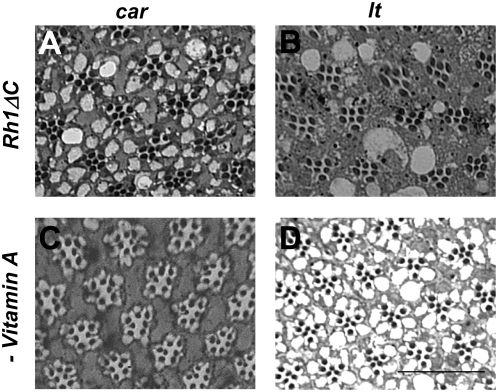
The light-dependent retinal degeneration that is observed in *car* and *lt* flies is rescued by the removal of rhodopsin or by preventing its endocytosis. Cross sections (1 µm) of retinas from (A) *car;;Rh1*Δ*356* flies, (B) *lt;Rh1*Δ*356* flies, (C) *car* flies raised on vitamin A-deficient media and (D) *lt* flies raised on vitamin A-deficient media. Flies were exposed to 7 days of constant room light. Eyes were fixed and embedded as described in Materials and Methods. Scale bar, 20 µm.

One model to explain the light-dependent nature of cell death in the granule group mutants is the fatal accumulation of Rh1 in the endosomal system. To confirm that Rh1 endocytosis following light-stimulation is necessary for cell death in these mutants, we examined the effect of Rh1 C-terminal deletion. Rh1 C-terminus is phosphorylated at a series of serine and threonine residues by the Rh1-kinase [24]. Elimination of these residues by C-terminal deletion (Rh1Δ356) or replacement with alanine prevents Arr1 [6] as well as Arr2-mediated endocytosis [12]. However, as determined in earlier studies, flies carrying this rhodopsin variant do not undergo retinal degeneration [12],[24]. We introduced a transgene encoding Rh1Δ356 in *car;;ninaE^I17^* and *lt;ninaE^I17^* mutant backgrounds. *ninaE^I17^* is a null allele of the gene coding for Rh1 and thus these flies did not express any full length Rh1. Expression of Rh1Δ356 rescued the light-dependent retinal degeneration observed in *car* and *lt* mutants (Figure 2A and 2B). These results suggest that light-induced Rh1-endocytosis causes retinal degeneration in granule group mutants.

We hypothesized that Rh1 endocytosis leads to its buildup due to lysosomal degradative defects in granule group mutants and accumulation of Rh1 might be the cause of photoreceptor cell death. To address this hypothesis, we investigated the effect of reducing the amount of Rh1 in photoreceptor cells. Rh1 consists of the protein moiety called opsin and a covalently attached chromophore, 11-*cis* 3-hydroxyretinal, that is derived from vitamin A. It has been demonstrated that raising flies on vitamin A deficient media reduces Rh1 levels to <3% of normal [25],[26]. We reasoned that, reducing the total amount of Rh1 by vitamin A deprivation would result in internalized Rh1 levels that are more manageable by the partially functional endo-lysosomal system and would thus prevent Rh1 accumulation. As has been previously reported for other backgrounds [12], Vitamin A deprived *car* and *lt* flies had smaller rhabdomeres. However, exposure to constant light for seven days did not result in any discernable change in rhabdomere structure that is characteristic of retinal degeneration (Figure 2C and 2D). Thus these two lines of evidence suggest that, reducing endosomal buildup specifically of Rh1 by diminishing its endocytosis or by reducing its protein levels rescues photoreceptor cell death.

### Light-Dependent Rh1 Endocytosis Leads to Its Presence in the Late Endosomes

To study the dynamics of Rh1 trafficking following endocytosis, we examined Rh1 distribution in the endocytic system. Because of the evidence of involvement of the late endosomal system in retinal degeneration, we focused on colocalization of Rh1 with Rab7, the widely used late endosomal marker [17], [27]–[30]. In dark-adapted wild type flies Rh1 was observed in crescent-shaped staining at the base of the rhabdomeres of the outer (R1-R6) photoreceptor cells (Figure S1). The Rab7 staining was solely observed in the cell body and was absent in the rhabdomeres. A few Rh1-positive vesicles were also observed in the cell body (at a frequency of 4-6 vesicles per 100 ommatidia). Localization of Rh1 in dark-raised *norpA*, *car,* and *lt* flies was similar to wild type, indicating that these mutations do not affect trafficking of Rh1 to the rhabdomere after its biosynthesis (Figure S1). As in wild type flies, Rh1-positive vesicles were also observed in these backgrounds in the cell body with similar frequency.

**Figure 3:**
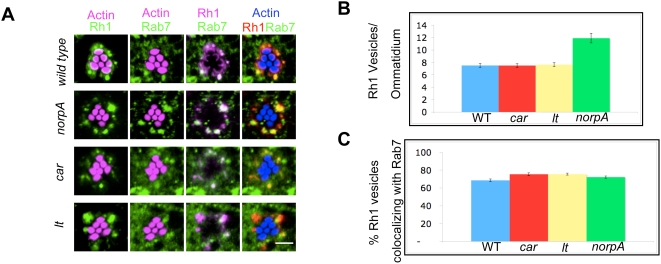
Light-induced endocytosis of rhodopsin and its presence in late endosomes. (A) Indirect immunofluorescence of whole-mounted retinas stained for Actin, Rhodopsin (Rh1) and Rab7. Wild type control (*w*), *norpA*, *car* and *lt* flies subjected to 24 hours of continuous light treatment and the retinas were stained as described in Materials and Methods. Light-exposure results in endocytosis of Rh1 from the rhabdomere membrane into the cell body, where a majority of it co-localizes with Rab7. We hypothesize that the poor rab7 staining observed in the granule group mutants is due to rab7GDP being sequestered in a complex with GDI due to the lack of GDP-exchange activity. Scale bar, 5 µm. (B) Quantitation of Rh1-positive endocytic vesicles in white-eyed wild type control (WT), *car, lt* and *norpA* flies after treatment with constant light for 24 hours. Number of Rh1 puncta in the cell body were counted in a confocal section as described in Materials and Methods and divided by the total number of corresponding ommatidia ascertained by Actin staining. Rh1-positive vesicles for WT control, 7.5120±0.326; for *car* flies, 7.4990±0.299; for *lt* flies, 7.6450±0.311 and for *norpA* flies, 11.920±0.77 (n = 140–160 ommatidia). Data are represented as Mean±SEM. (C) Quantitation of Rh1-positive late endosomes in wild type (WT), *car*, *lt*, and *norpA* flies. Number of Rh1-Rab7 double positive puncta were counted in confocal sections as above and divided by the total number of Rh1-positive puncta to calculate the percentage of Rh1-positive vesicles that are Rab7-positive. Percentage of Rh1-positive vesicles that are Rab7-positive for WT controls, 68.59±0.01; for *car* flies, 75.39±0.02; for *lt* flies, 75.50±0.01; for *norpA* flies, 72.59±0.01 (n = 981–1606 Rh1-positive vesicles). Data are represented as Mean±SEM.

We then examined Rh1-internalization in wild type flies following one day of light treatment. After photoactivation, endocytosed Rh1 was present in the cell body of the photoreceptors as large endocytic vesicles (Figure 3A), with 69%, of these Rh1-positive vesicles being Rab7-positive (Figure 3C). Colocalization of internalized Rh1 with late endosomal markers has been previously reported [31]. Thus, our results align with the previously published data that endocytosed Rh1 localizes in the late endosomes.

In *norpA*, *car*, and *lt* photoreceptors a significant number of Rh1-positive vesicles were formed after 1 day light-treatment (Figure 3A and 3B). More Rh1 positive vesicles are detected in *norpA* photoreceptors possibly due to the faster rate of Rh1 endocytosis observed in these flies. As in wild type flies, a majority of these vesicles from all three mutant backgrounds were Rab7-positive indicating their late endosomal nature (Figure 3C). Together, these data indicate that Rh1 is effectively delivered to the late endosomes in the granule group mutants as well as in *norpA*.

### Persistent Presence of Rh1 in Late Endosomes Indicates Defects in Its Degradation

Intracellular vesicle transport is a dynamic process that relies on highly orchestrated membrane traffic between adjacent organelles [32],[33]. Though, co-localization experiments as above provide a reasonable snapshot in time of the Rh1 vesicular traffic, they might not be truly reflective of changes in the trafficking kinetics due to perturbation in Rh1 influx or efflux. We therefore, explored the fate of Rh1 after it is delivered to the late endosomes by carrying out pulse-chase experiments. After internalization, Rh1 persists in the endosomes for approximately 13 hours in wild type flies ([31] and data not shown). We exposed flies for 2 days of continuous light to ensure that rate of Rh1 internalization reaches a maximum level. We then shifted the light-treated flies to complete darkness for 13 hours to allow any internalized Rh1 to traffic through the endosomal system while preventing any new endocytosis. This procedure allows us to discern any changes in Rh1 trafficking past the late endosomal stage.

**Figure 4:**
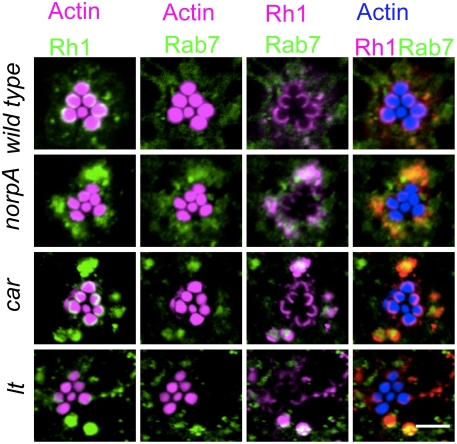
Rhodopsin accumulates in the late endosomes in *norpA* and granule group mutants. Indirect immunofluorescence of whole-mounted retinas stained for Actin, Rh1 and Rab7. Wild type control (*w*), *norpA*, *car* and *lt* flies were subjected to 48 hours of constant room light-treatment followed by 13 hours in complete darkness. The retinas were dissected and stained as described in Materials and Methods. Endocytosed Rhodopsin is cleared from the cytoplasm of retinas in wild type flies. Rhodopsin persists in Rab7-positive vesicles in *norpA*, *car* and *lt* flies. Scale bar, 5 µm.

In wild type flies that are subjected to the light/dark treatment as described above Rh1 was found at the base of the rhabdomere and the cytoplasm is mostly devoid of any Rh1-positive vesicles (Figure 4). There were a few Rh1/Rab7-double positive vesicles found as in dark-raised flies. Some Rh1-positive, Rab7-negative vesicles were also observed (data not shown). Thus, in the absence of any additional endocytosis, all the internalized Rh1 completely exits Rab7-positive late endosomes in 13 hours in wild type flies. On the contrary in *norpA* flies subjected to similar light treatment, Rh1-positive vesicles showed a very different behavior. In these flies some Rh1 immunoreactivity reappeared in the rhabdomere (Figure 4). However, Rh1-positive vesicles persisted in the cytoplasm. These vesicles took an amorphous shape uncharacteristic of the Rh1 vesicles in light-treated flies. Moreover, the persistent Rh1 vesicles stained positive for Rab7, indicating their late endosomal nature. The observed Rh1 accumulation in *norpA* flies is not due to global turning-off of cellular process as a result of pro-death signaling. The photoreceptors of *norpA* mutants subjected to continuous light treatment for 2 days followed by a shift to complete darkness for 3 days do not display any retinal degeneration and resemble the dark-raised *norpA* control flies (Figure S2). Thus cell death is not elicited after 2 days of light-exposure, a condition used in our experiments.

A similar defect in exit from the late endosomes was also illustrated in *car* and *lt* mutants (Figure 4). In these mutants, like in *norpA*, persistent Rh1-positive vesicles were formed that took an amorphous shape. Since these persistent vesicles stained for Rab7 and formed in mutants that are defective in late endosome to lysosome trafficking, we postulate that persistent vesicles represent the accumulated Rh1 that fails to be degraded in the 13 hour time period. These data indicate that the increased endocytosis of Rh1 (as in *norpA*) or decreased lysosomal delivery (as in *car* and *lt*) results in the endocytosed Rh1 trapped in the late endosomal stage.

### Internalized Rh1 Protein Is Not Completely Degraded in *norpA*

**Figure 5:**
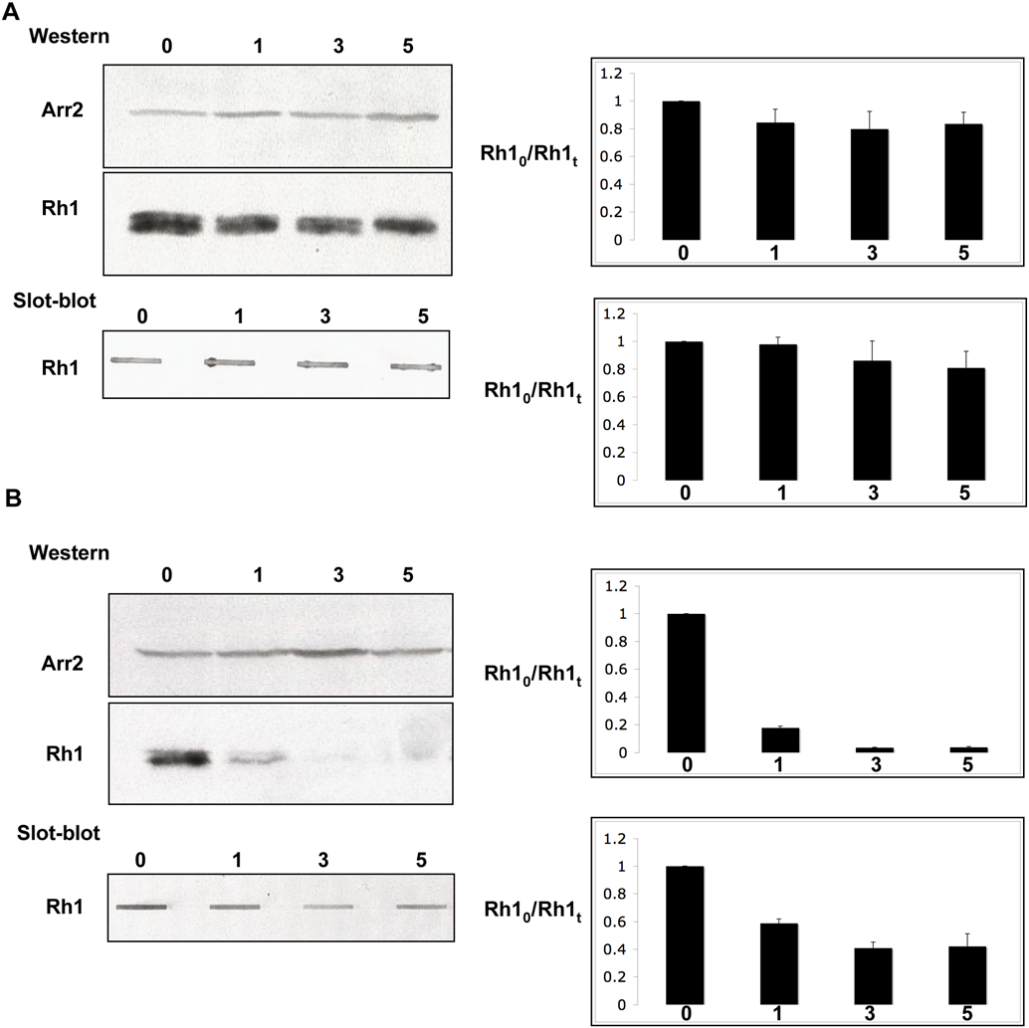
Rhodopsin is not degraded in light-treated *norpA* flies. (A) Head lysates were prepared from white-eyed control flies exposed to light for indicated time period and subjected to Western and Slot-Blot analysis. For Western blots, head lysates were fractionated by SDS-PAGE and probed with antibodies against Rh1 and Arr2. The slot-blot analysis was carried out as described in Materials and Methods and blots were probed with antibodies directed against Rh1. The steady-state level of Arr2, another photoreceptor-specific protein, is used as a loading control. (B) Head lysates from *norpA* flies exposed to light were subjected to Western and slot-blot analysis. Western analysis reveals that the Rh1 protein level drastically decreases with increasing light exposure. Contrary to this observation, slot-blot analysis reveals that Rh1 persists in light-exposed *norpA* flies. The densitometry data are represented as Mean±SEM. Results here show data from three independent experiments.

We then examined the steady-state level of Rh1 in light-exposed wild type and *norpA* flies. In wild type control flies the level of Rh1 remained constant as the function of light exposure (Figure 5A). Western blot analysis of Rh1 in *norpA* flies demonstrate that in dark-raised *norpA* flies Rh1 is expressed at wild type levels. Exposure to room light for 1, 3 or 5 days causes Rh1 steady state level to decrease rapidly. After one day of light exposure, the amount of Rh1 reduced to ∼20% of Rh1 level in dark-raised *norpA* flies (Figure 5B). The level of Rh1 falls subsequently to ∼5% of the original with increased light exposure.

We repeated these experiments using slot blots rather than westerns. Slot-blot analysis takes into account total protein content rather than solubility whereas western blot analysis detects only the soluble protein that can traverse the gel matrix. Interestingly, slot-blot analysis of protein extracts, demonstrated that in light-exposed *norpA* photoreceptors ∼40% of the original Rh1 protein was retained even after 5 days (5B). Rh1 in *norpA* flies remains undetectable by western-blot analysis possibly indicates that rapid endocytosis of large amount of Rh1 leads to its accumulation at extremely high local concentration, resulting in insolubility. The observation that total Rh1 level does not change appreciably even after prolonged light-treatment, is consistent with our conclusion that lack of protein degradation arises due to decreased late endosome to lysosome trafficking in *norpA*.

## Discussion

**Figure 6:**
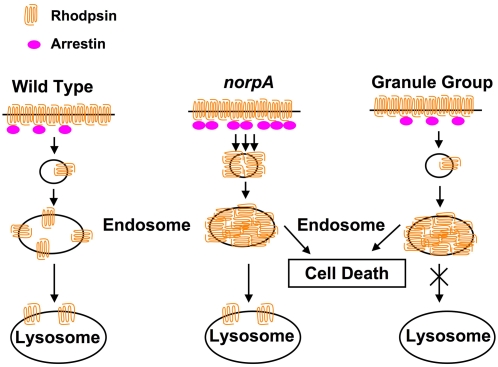
A model for light-dependent retinal degeneration. In wild type flies, Rh1 is endocytosed upon light-activation. The endocytosed Rh1 undergoes regular lysosomal turnover. In *norpA* flies, a large number of stable Rhodopsin-Arrestin complexes are formed which undergo rapid endocytosis. Massive endocytosis of Rhodopsin overwhelms the endocytic machinery and this rhodopsin fails to undergo degradation by the lysosomes and hence accumulates in the late endosomes. Endosomal accumulation of Rhodopsin triggers cell death by unknown mechanisms. This condition is simulated in granule group mutants that are defective in lysosomal delivery of endocytosed cargo. In these mutants, Rh1 internalization is similar to that in wild type but Rh1 accumulates in late endosomes as a result of defective trafficking. This accumulation of Rhodopsin also leads to photoreceptor cell death.

Proper regulation of rhodopsin endocytosis is essential for photoreceptor cell viability. In wild type photoreceptors, transient interaction of the major arrestin (Arr2) with Rh1 results in the deactivation of photoresponse. Light-dependent phosphorylation of Arr2 prompts its release from Rh1 [7]. However, in *norpA* photoreceptors, stable Rh1-Arr2 complexes are formed because of absence of Arr2 phosphorylation [12]. This causes endocytosis of Rh1 due to Arr2's ability to interact with the AP-2 adaptor protein and engage the endocytic machinery [13]. Here we demonstrate that rapid endocytosis of Rh1 in *norpA* leads to its accumulation in the late endosomes. It has been previously reported that Rh1 is a very abundant protein in the outer photoreceptor cells [34],[35]. Based on our current data we hypothesize that sudden endocytosis of a majority of Rh1 such that the lysosomal system is saturated, results in its accumulation in the late endosomes. We can induce Rh1 accumulation by causing slower lysosomal turnover, as in the granule group mutants, and this simulates gradual, light-dependent retinal degeneration in *norpA*. Reducing endocytosis prevents Rh1 buildup and rescues retinal degeneration. Similarly, preventing Rh1 accumulation in granule group mutants also rescues photoreceptor cell death. These data are consistent with a model that photoreceptor cell death is induced by the accumulation of Rh1 in the endosomes (Figure 6). Our results also indicate that Rh1 is not degraded in *norpA*; instead it becomes insoluble and undetectable on western blots, most likely due to formation of high-molecular weight aggregates.

The granule group mutants used in this study are mild loss-of-function alleles [36] and are viable. Severe phenotypes, including lethality, are observed in null alleles [37] or when two partial loss-of-function alleles are combined [36],[38] supporting the idea of an essential role played by these genes in general endocytic trafficking. Since null alleles of the granule group genes result in lethality, our analysis was restricted only to the available viable alleles of these genes. Our analysis of multiple genes affecting late endosomal/lysosome trafficking rules out the light-dependent degeneration as an artifact. Despite their impact on endocytosis, we believe that the photoreceptor cell death observed in the granule group mutants is specifically due to Rh1 accumulation and not because of trafficking impairment of other cargo molecules. These mutants do not display any retinal degeneration in continuous darkness—a condition in which Rh1 endocytosis is minimal. Absence of any photoreceptor degeneration in darkness discounts the idea that cell death is due to an effect on “housekeeping” endocytosis in these mutants. We also demonstrate that the photoreceptor death is rescued when the C-terminal region of Rh1 is deleted. This prevents endocytosis specifically of Rh1 and should not affect any other cellular process. Our results of vitamin A deprivation experiments also rule out the possibility of any pleiotropic effects of these mutations as the cause of retinal degeneration. Vitamin A is solely required for visual pigment biosynthesis and does not play any role in cellular physiology in *Drosophila* [25]. Rescue of light-dependent retinal degeneration of granule group mutants by vitamin A deprivation suggests that reduction of Rh1 protein level is sufficient to prevent retinal cell death.

It is also possible that a second molecule capable of triggering a pro-cell death pathway from endosomes is internalized with Rh1. Thus preventing endocytosis of Rh1 or its accumulation may prevent cell death signaling by this molecule. Arr2, a molecule speculated to be involved in pro-death signaling [6],[9],[12] was absent on persistent vesicles in *norpA* and *car* flies (data not shown). Similarly, we have not been able to detect ubiquitylation of Rh1 (P.J. Dolph, unpublished data). Currently we cannot elucidate the identity (Rh1 or other protein) of the molecule that triggers cell death. Regardless of the identity of the pro-death molecule, based on our data we conclude that timely degradation of Rh1 by fully functional lysosomal machinery is essential for maintaining photoreceptor viability.

Our finding, that failure to clear accumulated Rh1 causes cell death, has relevance to human disease. Autosomal Dominant Retinitis Pigmentosa (ADRP) is a retinal degenerative disorder and is a common cause of blindness affecting one in 3000 people (RetNet, http://www.sph.uth.tmc.edu/Retnet/). Mutations in the human rhodopsin that affect its folding, trafficking and activity are the most commonly encountered causes of retinal degeneration in afflicted patients. Missense mutations in the opsin gene affecting the R135 and K296 residues of the protein product cause ADRP and result in accumulation of Rhodopsin-Arrestin complexes in the photoreceptor cell [10],[11]. The R135 mutant rhodopsin is noted to form stable complex with arrestin and undergo endocytosis resulting in aberrant endocytic vesicles in HEK cell culture system [11]. Similarly, the K296E rhodopsin is observed to bind the visual arrestin with high affinity. This abnormal interaction is demonstrated to have pathological consequences in the retina. Besides this, the stable rhodopsin and arrestin complexes are shown to mislocalize and accumulate in the inner segments of rod photoreceptors of the mouse model of ADRP. Thus the accumulation of rhodopsin might trigger cell death by a similar mechanism as in *Drosophila norpA* and granule group mutants.

In this work, using the anti-Rh1 antibody, we observe that Rh1 staining at the base of the rhabdomeres in a crescent-shaped pattern in adult flies. Similar results with adult flies have been reported previously [6],[39],[40]. This is in contrast to earlier reports where Rh1 staining was uniformly present in the entire rhabdomere [5],[8],[13]. The different results could be attributed to the differences in immunolocalization methods. Previous studies have used cryosectioning followed by immunostaining, while this study utilizes whole-mount immunostaining to localize Rh1. It is possible that the crescent-shaped staining of Rh1 in whole-mount samples is observed due to inaccessibility to rhabdomeric Rh1 as a result of highly organized and densely packed microvillar structure in adult fly eyes. The uniform distribution of GFP-tagged Rh1 throughout the rhabdomere in whole-mounted retinas supports this explanation (Figure S3). Regardless of the observed differences in the rhabdomeric Rh1 staining patterns, our ability to detect persistent Rh1-positive vesicles in the photoreceptor cell body leads us to propose that cytoplasmic accumulation of Rh1 in certain genetic backgrounds results in their light-dependent retinal degeneration. We also observe that Rh1 immunoreactivity is lost as a function of light-exposure on the western-blots in *norpA* flies. However, slot-blot analysis reveals that Rh1 persists in light-exposed *norpA* flies. The observed differences in the results obtained by two techniques can be attributed to the change in protein solubility, most likely due to protein aggregation and formation of high molecular weight protein complex. We demonstrate that Rh1 in *norpA* accumulates in late endosomes. Late endosomes are acidic compartments [41] and Rh1 aggregates under acidic conditions (P. J. Dolph, unpublished). We speculate that under extremely high concentration and acidic pH, such as those presented in *norpA*, Rh1 can aggregate and form a high molecular weight complex. This complex fails to traverse the gel matrix in the SDS-PAGE and hence remains undetectable on the western-blot while it can be easily detected by slot-blot analysis. A single base-substitution at the codon position 23 in the human opsin gene (P23H) is the most common cause of ADRP in American patients [42]. P23H rhodopsin is extremely prone to aggregation and forms high-molecular weight complexes [43],[44]. Similarly, the aforementioned K296E mutation is also shown to become insoluble and form aggregates in cell culture [44]. This bears remarkable resemblance with some neurodegenerative disorders such as Alzheimer's disease, Huntington's disease, Spinocerebellar ataxias and Prion diseases, where protein misfolding, aggregation and cytoplasmic accumulation are the implicated causes of cell death [45]. It has also been demonstrated that late endosomal accumulation of cholesterol [46], prion [47] and amyloid β-protein [48] is associated with Niemann-Pick type C disease, Scrapie and Alzheimer's Disease respectively. Therefore it is tempting to speculate that protein accumulation results in neuronal death by utilizing similar pathways in *norpA* and neurodegenerative diseases.

Our results raise one intriguing question. That is, how can vesicular accumulation of Rh1 elicit pro-cell death signaling? The importance of proper regulation of Rh1 endocytosis in maintaining photoreceptor cell viability is increasingly appreciated [6],[13]. But the precise mechanisms regulating the pro-cell death signaling pathways and their interconnection with endocytosis is not well understood. Conventional developmental apoptosis involving caspase-activation plays a marginal role in *norpA* retinal degeneration [49]. Moreover, induction of non-apoptotic, cell death by arrestin2 and the heptahelical neurokinin1 receptor is also reported [50]. It is plausible that persistent cytoplasmic presence of Rh1 activates photoreceptor cell death by a similar mechanism. We speculate that a component innate to the endolysosomal system plays a crucial role in regulating the cell death signals emanating from the endosomes. Accumulation of Rh1 is sensed by this component, which can then engage the cell death machinery to execute cell death in the retina. Failure of proper protein degradation and resultant subsequent accumulation of proteins is a well-recognized cause of cell death in many neurodegenerative disorders [51]. Proteins involved in late endosome-lysosome trafficking also play a role in lysosomal degradation in autophagy [52]. Interestingly lysosomal clearance of accumulated cytoplasmic proteins by induction of autophagy is also reported to have protective effect on cells [53],[54]. We thus hypothesize that increasing the lysosomal turnover of endocytosed Rh1 should rescue the photoreceptor cell death in *norpA* and the granule group mutants.

Our work shows that lysosomal turnover of Rh1 plays a vital role in photoreceptor health and causing Rh1 buildup in late endosomes initiates pro-death signaling. The novel nature of this trigger and its relevance to human disease warrants further analysis of the endolysosomal system and its connection to the cell death machinery. *Drosophila* photoreceptor neurons have served as an ideal genetic and cellular platform to model human retinal and neurodegenerative disorders. Detailed analysis of the endolysosomal system in these cells should lend valuable insights into the mechanism of induction of cell death by protein accumulation and lysosome dysfunction.

## Materials and Methods

### *Drosophila* Stocks

The *Drosophila melanogaster* stocks *carnation (car^1^), carmine (cm^1^)* and *light (lt^1^)* were obtained from the Bloomington stock center, Indiana. The *norpA^EE5^* mutation is induced by EMS and has been previously described [12],[13]. All flies were crossed into a *white (w^1118^)* background in order to completely eliminate screening pigments in the compound eye. Thus *w^1118^* served as a wild type control in all our experiments. The Rhodopsin C-terminal deletion mutant (*Rh1Δ356*) has been previously described [24]. The fly stock expressing GFP-tagged Rh1 was a kind gift from Dr. Joseph O'Tousa. For light-exposure experiments, the flies were exposed to room light that was 7.5 µmol/m^2^/sec of visually active radiation (38–710 nm) as detected by a quantum sensor.

### Histological Fixation and Sectioning

Flies were reared on cornmeal-molasses medium. Vitamin-A-deficient medium was prepared and flies were deprived of vitamin A as described previously [12]. Dark-adapted flies were exposed to constant light at room temperature. Heads of the light-treated flies were bisected and immersed in ice-cold 2% glutaraldehyde. An equal volume of 2% osmium tetroxide was added and incubation carried on ice for 30 minutes. The glutaraldehyde/osmium tetroxide mixture was removed and the eyes were washed with 0.1 M phosphate buffer followed by treatment with 2% osmium tetroxide for 1.5 hours on ice. The eyes were then dehydrated with increasing concentration of ethanol (30%, 50%, 70%, 90%) for 10 minutes each on ice followed by treatment with 100% ethanol at room temperature for 10 minutes twice. This was followed by two propylene oxide (Electron Microscopy Sciences) washes for 10 minutes each and incubation in 50% propylene oxide/50% Durcupan (Fluka) overnight at room temperature. This mixture was then replaced with 100% durcupan and incubated for 4 hours. Eyes were then embedded in molds and cured at 75°C overnight. Cross sections (1 µm) were cut using a Sorvall ultra microtome MT-1 (Sorvall, CT). The sections were stained with toluidine blue and borax and observed on a Zeiss Axioplan 2 microscope using a 63X/1.4 NA oil immersion objective. Digital images were captured using a Optronics DEI-750 camera (Optronics) and MetaVue (Universal Imaging) software.

### Antibodies and Immunohistochemistry

Anti-rab7 antibody was generated in rabbits (Cocalico Biologicals, PA) against the unique C-terminal 45 amino acids of *Drosophila* Rab7 protein. This antibody recognized a single 27 kDa protein in homogenates prepared from wild type fly heads. To further confirm the specificity of this antibody, we expressed GFP-Rab7 and GFP-Rab5 fusion proteins in separate fly lines using the GMR-Gal4/UAS system. This antibody recognized the GFP-Rab7 fusion protein but did not recognize the GFP-Rab5 protein in fly head homogenates when assayed by western blot analysis. Other reported antibodies for the late endosome-specific proteins of *Drosophila,* namely Anti-Dor, Anti-Hook and Anti-LBPA yield inconsitent or no signal in photoreceptors (Anti-Dor; this study) and (Anti-Hook and Anti-LBPA; [31]). Retina from adult flies was dissected away from the cornea and the underlying brain parts and was prepared for whole-mount immunostaining essentially as described in [6]. For dissections in darkness, eyes from dark-adapted flies were dissected under safelight illumination using 1A and GBX-2 filters (Kodak). Illumination with these lights did not elicit any ERG response. Isolated retinas were incubated in diluted primary antiserum overnight at 4°C. The antibodies were diluted in 1X PBS+0.3% Triton X-100 (PBX)+10% Fetal Calf Serum (FCS). After 3 washes with PBX for 5 minutes each, the tissue was incubated in secondary antibodies diluted in PBX+FCS for 12–14 hours at 4°C. After two brief washes with PBX at room temperature and a prolonged, overnight wash at 4°C, the eyes were rinsed with 1X PBS for 3 times. The eyes were mounted in 50% Glycerol/1 X PBS/0.5% n-Propyl Gallate and viewed over Leica TCS SP confocal laser-scanning microscope (Leica Microsystems, Heidelberg, Germany). The primary antibodies were anti-Rh1 (1:50) (Developmental Studies Hybridoma Bank, Iowa city, IA), anti-Rab7 (1:100) (this study). For visualizing rhabdomeres, F-actin was stained using Rhodamine- or Alexa-568-conjugated phalloidin (Molecular Probes) (5 U/mL) added to the diluted primary antibodies. Secondary antibodies were anti-mouse or anti-rabbit labeled with Alexa-488 or Alexa-647 (1:300) (Molecular Probes), Cy5-conjugated anti-mouse (1:200) and FITC-conjugated anti-rabbit (1:200) (Jackson ImmunoResearch, PA).

The captured images were processed with NIH ImageJ and/or Adobe Photoshop. Whenever possible, the images were made colorblind-compatible. The image processing conformed to the guidelines laid down by Rossner and Yamada [55]. For whole-mount immunostaining, retinas from a minimum of five flies were isolated and processed as above. For quantifying Rh1 vesicles, cytoplasmic spherical structures staining positive for Rh1 were counted in 140–150 ommatidia per experiment.

### Biochemistry

Fly heads were homogenized in Laemmli 1X SDS loading buffer (SDSLB). The lysate was subjected to SDS-PAGE followed by western analysis as described in [12] using antibodies against Rhodopsin (4C5, 1:5000) and Arrestin-2 [5] (1:2000). The steady-state level of Arr2, a photoreceptor-specific protein, is used as a loading control.

For slot blot analysis, head lysate made in 1X SDSLB was adjusted to a final concentration of 1M Urea and applied to Bio Dot apparatus (BioRad, CA) and transferred to OptiTran BA-S 85 supported nitrocellulose membrane (Whatman, Germany) as per apparatus manufacturer's instructions. The membrane was incubated in 0.2 N sodium hydroxide for 30 minutes and washed three times with 1X PBS+0.1 % Tween-20. Blocking and antibody incubations were carried out as in western analysis. The blots were subjected to densitometry analysis using the LabWorks Imaging and Analysis software (UVP, Inc., Upland, CA). The intensity of Rh1 band for a particular time period (t) was divided by the intensity of Rh1 band corresponding to lysates prepared from dark-adapted (Rh1_0_) flies to arrive at the Rh1_t_/Rh1_0_ value. The Rh1_t_/Rh1_0_ values from three independent experiments were used for analysis.

## Supporting Information

Figure S1 Rhodopsin immunostaining is detected at the base of the rhabdomere. Indirect immunofluorescence of whole-mounted retinas stained for Actin, Rh1 and Rab7. Wild type control (*w*), *norpA*, *car* and *lt* flies were raised in complete darkness and the retinas were isolated as described in Materials and Methods. Rh1 localizes to the base of the rhabdomeres in all the genotypes observed. Scale bar, 5 µm.
(4.49 MB TIF)

Figure S2 Cell death is not induced in *norpA* mutants after 2 days of light exposure. Confocal sections from the retinas isolated from 5 day old, dark-raised *norpA* flies (A) and *norpA* flies subjected to 2 days of constant room light exposure followed by a shift to complete darkness for 3 days (B). Retinas were prepared for whole-mount immunohistochemistry and stained for Actin as described in Materials and Methods. Scale bar, 5 µm.
(1.51 MB TIF)

Figure S3 Rhodopsin is localized throughout the rhabdomere. Confocal micrograph of whole mount retina isolated from the fly expressing GFP-tagged Rh1 driven by *rh1* promoter. The retinas were isolated and fixed as described in Materials and Methods and stained for Actin to visualize rhabdomeres. Rh1 was detected using fluorescence in the GFP channel of the microscope.
(4.36 MB TIF)
