# Supplementary figures and images for: Accumulation of Rhodopsin in Late Endosomes Triggers Photoreceptor Cell Degeneration
Source: PLoS Genet. 2009 Feb 13;5(2):e1000377. doi: 10.1371/journal.pgen.1000377 (PMC2633617; doi:10.1371/journal.pgen.1000377)

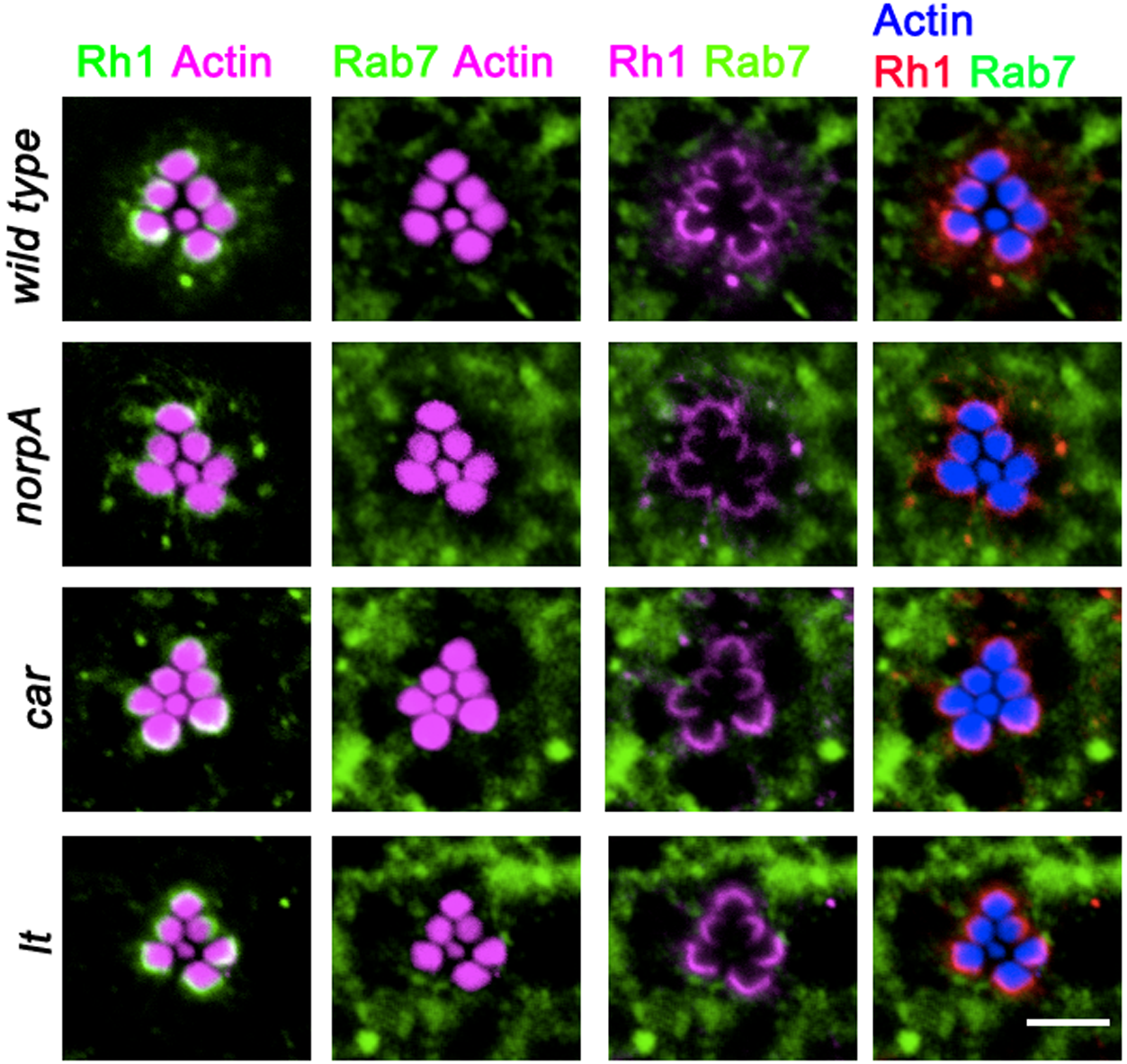

Supplement: Figure S1 — Rhodopsin immunostaining is detected at the base of the rhabdomere. Indirect immunofluorescence of whole-mounted retinas stained for Actin, Rh1 and Rab7. Wild type control (w), norpA, car and lt flies were raised in complete darkness and the retinas were isolated as described in Materials and Methods. Rh1 localizes to the base of the rhabdomeres in all the genotypes observed. Scale bar, 5 µm. (4.49 MB TIF) [file pgen.1000377.s001.tif]

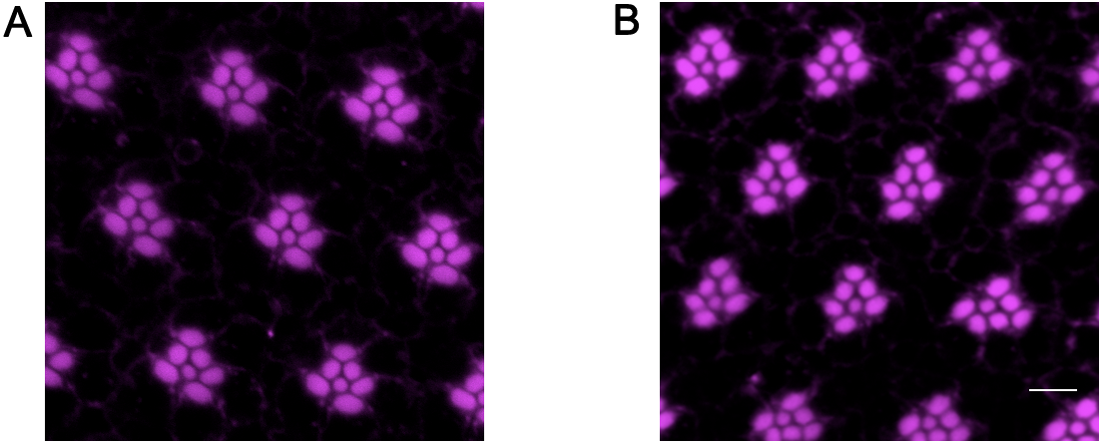

Supplement: Figure S2 — Cell death is not induced in norpA mutants after 2 days of light exposure. Confocal sections from the retinas isolated from 5 day old, dark-raised norpA flies (A) and norpA flies subjected to 2 days of constant room light exposure followed by a shift to complete darkness for 3 days (B). Retinas were prepared for whole-mount immunohistochemistry and stained for Actin as described in Materials and Methods. Scale bar, 5 µm. (1.51 MB TIF) [file pgen.1000377.s002.tif]

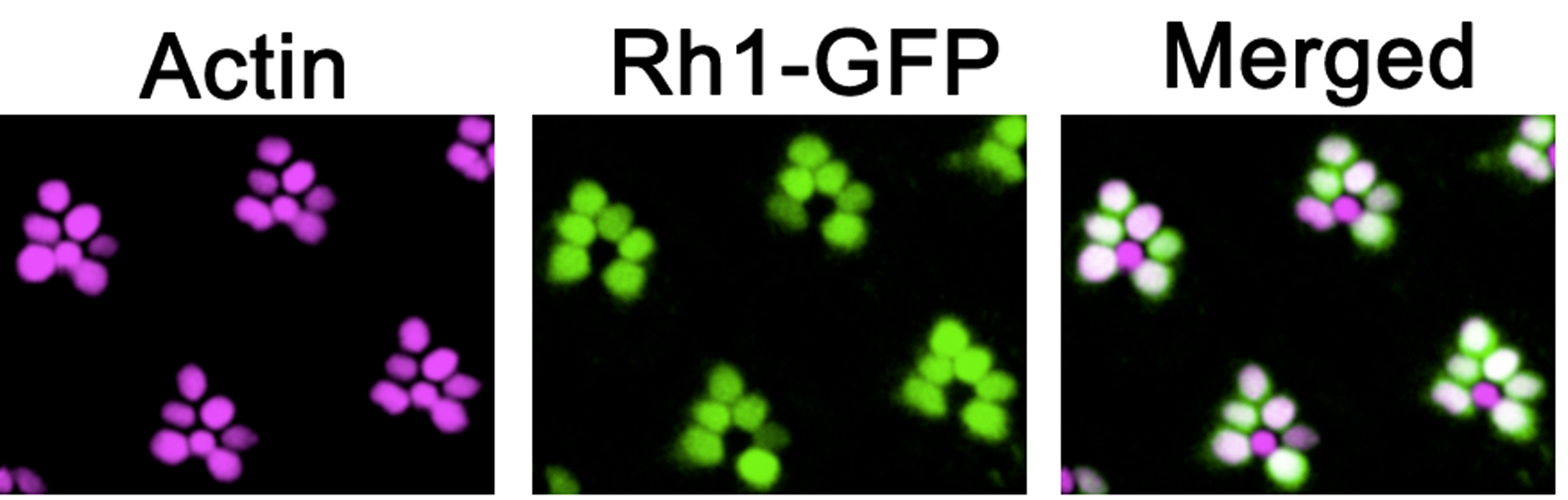

Supplement: Figure S3 — Rhodopsin is localized throughout the rhabdomere. Confocal micrograph of whole mount retina isolated from the fly expressing GFP-tagged Rh1 driven by rh1 promoter. The retinas were isolated and fixed as described in Materials and Methods and stained for Actin to visualize rhabdomeres. Rh1 was detected using fluorescence in the GFP channel of the microscope. (4.36 MB TIF) [file pgen.1000377.s003.tif]
